# A *de novo *complete *BRCA1 *gene deletion identified in a Spanish woman with early bilateral breast cancer

**DOI:** 10.1186/1471-2350-12-134

**Published:** 2011-10-11

**Authors:** Zaida Garcia-Casado, Ignacio Romero, Antonio Fernandez-Serra, Luis Rubio, Francisco Llopis, Ana Garcia, Pilar Llombart, Jose A Lopez-Guerrero

**Affiliations:** 1 Laboratory of Molecular Biology, Fundación Instituto Valenciano de Oncología, Valencia, Spain; 2 Unit of Genetic Counselling of hereditary cancer, Fundación Instituto Valenciano de Oncología, Valencia, Spain; 3 Service of General and Digestive Surgery, Fundación Instituto Valenciano de Oncología, Valencia, Spain; 4 Gynecology Service, Fundación Instituto Valenciano de Oncología, Valencia, Spain; 5 Psychology Unit, Fundación Instituto Valenciano de Oncología, Valencia, Spain

## Abstract

**Background:**

Germline mutations in either of the two tumor-suppressor genes, *BRCA1 *and *BRCA2*, account for a significant proportion of hereditary breast and ovarian cancer cases. Most of these mutations consist of deletions, insertions, nonsense mutations, and splice variants, however an increasing number of large genomic rearrangements have been identified in these genes.

**Methods:**

We analysed *BRCA1 *and *BRCA2 *genes by direct sequencing and MLPA. We confirmed the results by an alternative MLPA kit and characterized the *BRCA1 *deletion by Array CGH.

**Results:**

We describe the first case of a patient with no strong family history of the disease who developed early-onset bilateral breast cancer with a *de novo *complete *BRCA1 *gene deletion in the germinal line. The detected deletion started from the region surrounding the *VAT1 locus *to the beginning of *NBR1 *gene, including the *RND2*, Ψ*BRCA1*, *BRCA1 *and *NBR2 *complete genes.

**Conclusion:**

This finding supports the large genomic rearrangement screening of *BRCA *genes in young breast cancer patients without family history, as well as in hereditary breast and ovarian cancer families previously tested negative for other variations.

## Background

Breast cancer is the most common cancer among women, excluding non-melanoma skin cancers, and constitutes, after lung cancer, the second leading cause of cancer deaths in women. According to the American Cancer Society, about 1.3 million women will be diagnosed with breast cancer annually worldwide, and about 465,000 will die from this disease [1]. About 5-10% of all breast cancers are estimated to be hereditary, and germline mutations in the tumor-suppressor genes *BRCA1 *(MIM#113705) and *BRCA2 *(MIM#600185) are found in a proportion of this group [2,3]. Family history of breast and ovarian cancer, besides breast cancer bilaterality, early-onset breast cancer and ethnicity, constitute the basic criteria for identifying cases affected by *BRCA1 *or *BRCA2 *mutations. However, a negative family history does not exclude the presence of a germline mutation in these genes; in fact, in unselected populations, the estimated prevalence of *BRCA1 *mutations in medullary and triple negative breast cancers is about 18% before age 50 [4-8].

Most of the reported *BRCA1 *and *BRCA2 *mutations are characterized by deletions, insertions, nonsense mutations and splice variants that result in a truncated protein. Nevertheless, an increasing number of large genomic rearrangements (LGRs), not detectable by current PCR-based methods, have been identified in these genes [9], mainly due to the development of the multiplex-ligation-dependent probe amplification (MLPA) procedure that allows the screening of LGRs in a large number of samples. Prior to MLPA, LGRs were analysed by different approaches such as Southern-blot, long-range PCR, fluorescence in situ hybridization-based methods and real-time PCR. LGRs in *BRCA1 *are responsible for between 0 and 27% of all *BRCA1 *disease-causing mutations identified in different populations [9] whereas in the case of *BRCA2 *these rearrangements are rare, except for a Portuguese population with a founder rearrangement [c.156_157insAlu (NG_012772.1:g.8686_8687insAlu)] that explains more than a quarter of *BRCA *mutations [9,10].

Identification of *BRCA *mutation carriers allows non-directive clinical decisions to be made [11], in the management of high lifetime risk of breast and ovarian cancer including follow-up, prophylactic mastectomy and salpingo-oophorectomy. Furthermore, mutations in *BRCA *have been shown to be predictive of a good response to certain treatments. For example, in a neoadjuvant setting with cisplatin, an 83% pathologic complete response rate in *BRCA1 *breast cancer carriers has been reported [12]. Furthermore, treatment with the new Poly(ADP)-Ribose Polymerase inhibitors, still under clinical development, has shown promising results in targeting the BRCA-related homologous recombination pathway [13-16].

We report the first case of a patient with no strong family history of the disease, with a *de novo *complete *BRCA1 *gene deletion demonstrated by Array CGH that developed early-onset bilateral breast cancer.

## Methods

### Patients

A 39 year-old woman with bilateral metachronous breast cancer (at 28 and 37) was referred from the Service of Medical Oncology to the Unit of Genetic Counselling of hereditary cancer of our institution. After a pre-genetic work-up and a psychological interview, informed consent and a blood sample were obtained to perform direct sequencing and MLPA analysis of *BRCA1 *and *BRCA2*. Written consent to publish the information herein reported was also obtained from the patient.

**Figure 1:**
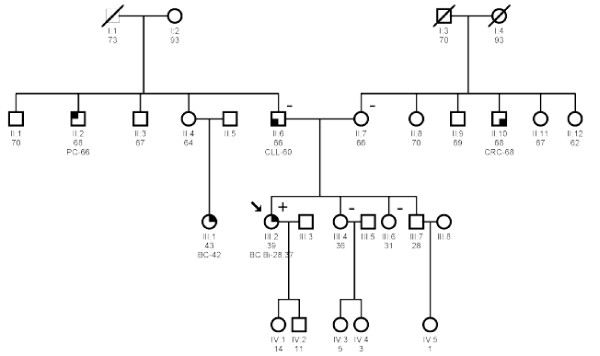
**Patient's family pedigree displaying breast cancer**. Black shading indicates individuals with cancer. The patient (proband) is individual III:2 and is arrowed. The current age and the age at diagnosis of cancers are indicated below. The genotypes (where DNA available for analyses) are shown as: - for wild-type homozygous, and + for heterozygous for *BRCA1 *complete deletion. PC: prostate cancer; CLL: chronic lymphocytic leukaemia; CRC: colorectal cancer; BC: breast cancer; Bi BC: bilateral breast cancer.

The case herein described presented no previous family history of breast or ovarian cancer (Figure 1), although the mother was surgery treated with a bilateral hysterectomy and oophorectomy at age 41 for a benign process. In 1998, our case (28 years-old) developed an infiltrating ductal carcinoma in the right breast (pT1cpN0M0) and was treated with quadrantectomy and axillary lymphadenectomy followed by external radiotherapy and interstitial brachytherapy, and 5 cycles of AC (cyclophosphamide, doxorubicin). Eight years later (at age 37), a medullary carcinoma was diagnosed in the contralateral breast (pT2pN0M0). On this occasion, the treatment consisted of 4 cycles of liposomal doxorubicin followed by external radiotherapy. In both cases the tumors expressed hormonal receptors and HER2.

As a *BRCA1 *LGR was identified, a prophylactic surgery was proposed, although finally the patient opted for a yearly follow-up consisting of mammography, transvaginal echography and measurement of serum levels of CA125.

Close relatives were invited to complete the segregation study and informed consent was obtained from the mother, father and two sisters. All of them were negative for this LGR. One brother, with a one year mentally retarded daughter, refused the genetic study.

### Mutation analysis of *BRCA1 *and *BRCA2*

Genomic DNA was isolated from peripheral blood samples with the automatic Magtration System 12GC and the Magtration-MagaZorb DNA Common kit (Precision System Science Co. Ltd.). DNA integrity was evaluated by the A260/A280 absorbance ratio with a Nanodrop-1000 (NanoDrop ND1000, NanoDrop Technologies, Wilmington, Delaware USA) spectrophotometer. Mutational screening of *BRCA1 *and *BRCA2 *genes was carried out by direct sequencing using the VariantSeq RSS000009249_03 and RSS000009432_04 assays (Applied-Biosystems, Foster City, USA), respectively, and specific primers to complete the sequence of both genes (primer sequences available upon request). DNA sequencing was performed directly on PCR purified products using the Big Dye terminator v3.1 sequencing kit (Applied-Biosystems, Foster City, USA). Capillary gel electrophoresis and data collection were carried out on an automated DNA sequencer ABI PRISM 3130XL (Applied-Biosystems). Sequence analyses were carried out with Seq-scape Software v2.6 (Applied-Biosystems). Mutation nomenclature is in accordance with the Human Genome Variation Society (HGVS) (http://www.hgvs.org/mutnomen/). The reference sequences used for *BRCA1 *and *BRCA2 *are NM_007294.2 and NM_000059.3 respectively from the NIH GeneBank (http://research.nhgri.nih.gov/bic/).

### LGR detection by Multiplex Ligation-dependent Probe Amplification (MLPA)

As no significant mutations were found by direct sequencing, *BRCA1 *LGR was quantified by MLPA using the P002 probe mix assay according to the manufacturer's instructions (MRC Holland). Once a positive result was obtained, a confirmatory analysis was independently performed with the *BRCA1 *P087 assay (MRC Holland). Amplified products were separated using an ABI PRISM 3130XL (Applera) genetic analyzer and interpreted using GeneMapper Software v4.0 (Applied-Biosystems). Quantitation of the results of fragment analysis was performed using the Excel software by calculating relative peak areas as described by the manufacturer (MRC Holland). Different normal control samples were used to normalize the allele dosage.

### Comparative Genome Hybridization (CGH) Array

Array CGH was performed once a complete deletion of *BRCA1 *was noticed by MLPA. Non-amplification labelling of DNA (direct method) was obtained following the 'Agilent Oligonucleotide Array-Based CGH for Genomic DNA Analysis' protocol Version 5.0 (Agilent Technologies, Palo Alto, California USA. p/n G4410-90010). Two μg of experimental and reference genomic DNA samples were fragmented in a restriction digestion step. Digestion was confirmed and evaluated by DNA 7500 Bioanalyzer assay. Cyanine 3-dUTP and cyanine 5-dUTP were used for the respective fluorescent labelling of test and reference-digested gDNAs using the 'Agilent Genomic DNA Labelling Kit PLUS' (Agilent p/n 5188-5309) according to the manufacturer's instructions. Labelled DNA was hybridized with the Human Genome CGH Microarray 244K (Agilent p/n G4423B-014693) containing 236,381 distinct biological features covering the human genome at an overall median probe spacing of 7.4 KB in Refseq genes. Arrays were scanned in an Agilent Microarray Scanner (Agilent G2565BA) according to the manufacturer's protocol, and data extracted using Agilent Feature Extraction Software 10.7.1 following the Agilent protocol CGH_107_Sep09, grid template 014693_D_F_20090929 and the QC Metric Set CGH_QCMT_Sep09. CGH data were analysed and visualized using the Genomic Workbench Standard Edition 5.0 (Agilent Technologies) software. The human reference sequence employed was the March 2006 NCBI36/hg 18 produced by the International Human Genome Sequencing Consortium.

### Exclusion of non-paternity

Once the absence of *BRCA1 *deletion was demonstrated in the parents of our case, a set of twelve polymorphic tetranucleotide repeats was analysed by three fluorescent multiplex PCR in order to exclude non-paternity [17]. Products were separated by capillary electrophoresis and analysed using GeneMapper Software v4.0 (Applied-Biosystems).

### Assignment of parental origin

To determine whether the mutation occurred on the maternal or paternal allele, we analysed seven *BRCA1 *intragenic polymorphisms (rs8176144, rs1799949, rs16940, rs1799966, rs3092987, rs8176235 and rs11654396) by direct sequencing.

### Copy Number Variation (CNV) Analysis

Copy number analysis of *ZFPM2 *was performed for the proband, her mother, father, two sisters and two controls, using the TaqMan^® ^Copy Number Assays (CNA) (Applied Biosystems). Three assays were selected for this purpose, one being located in proximity to the 5'-end of the *ZFPM2 *gene (HS06234652_cn), one near the 3'-end (HS02556672_cn), and the TaqMan Copy Number reference assay (*RNase P*), which is known to exist only in two copies in a diploid genome. Each DNA sample was analysed in quadruplicate. Reactions were performed according to the manufacturer's instructions and processed in an ABI 7500 Fast Real Time PCR System (Applied Biosystems). Data was collected by the SDS software (version 2.01; ABI) using the standard absolute quantification method. After the reaction, raw data was analysed using a manual cycle threshold (Ct) of 0.2 with the automatic baseline on, and then imported to the CopyCallerTM Software (version 1.0; ABI) for post-PCR data analysis. In the software, copy numbers were estimated using a maximum likelihood algorithm.

## Results

### Mutation analysis of *BRCA1 *and *BRCA2*

The analysis of the complete coding and exon-intron boundary sequences of *BRCA1 *and *BRCA2 *revealed no frameshift or missense mutations. Only a previously uncharacterized base change in the position IVS6+14 C > T (c.516+14 C > T according to HGVS nomenclature) of *BRCA2 *gene was found. Using bioinformatics tools, such as ESEfinder (http://rulai.cshl.edu/cgi-bin/tools/ESE3/esefinder.cgi?process=home) to identify exonic splice enhancer motifs; Splice Site Prediction by Neural Network (Berkeley Drosophila Genome Project) (http://www.fruitfly.org/seq_tools/splice.html), and Human Splicing Finder (http://www.umd.be/HSF/) to identify putative splice sites [18], this change was predicted not to affect splicing.

### MLPA analysis of *BRCA1 *and *BRCA2*

**Figure 2:**
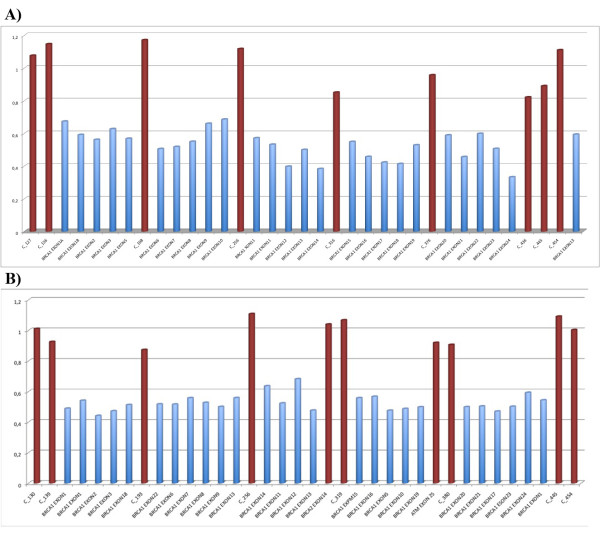
**MLPA analysis of the *BRCA1 *gene performed with the SALSA MLPA KIT P002 (panel A) and P087 (panel B) (MRC-Holland)**. Graph displaying the ratios between the relative peak areas for proband and controls. Grey and black bars correspond to *BRCA1 *and control probes respectively. The analysis revealed a 50% decreased amplification of the probes corresponding to *BRCA1 *gene.

Once direct DNA sequencing had detected no significant genetic variations, we proceeded to the LGR analysis of *BRCA1 *by MLPA (*BRCA1 *P002). The case showed an MLPA profile suggestive of a deletion involving the complete *BRCA1 *gene (Figure 2A). The analysis was confirmed by MLPA using the *BRCA1 *P087 kit with the same resulting profile (Figure 2B). Genetic counselling and testing of the *BRCA1 *deletion were offered to the patient's relatives (Figure 1). MLPA analysis of *BRCA1 *was carried out on blood samples from both parents and from the two proband's sisters, all showing negative results for *BRCA1 *deletion. In order to confirm paternity, a set of twelve polymorphic tetranucleotide repeats was analysed on DNA from the patient and both parents, the results were consistent with the reported paternity (data not shown). On the other hand, to determine whether the mutation occurred on the maternal or paternal allele, we analysed seven *BRCA1 *intragenic polymorphisms for both parents and compared their haplotypes with the haplotype of their daughter indicating that the *BRCA1 *deletion arose in the mother's germ cells (data not shown).

### Array CGH

**Figure 3:**
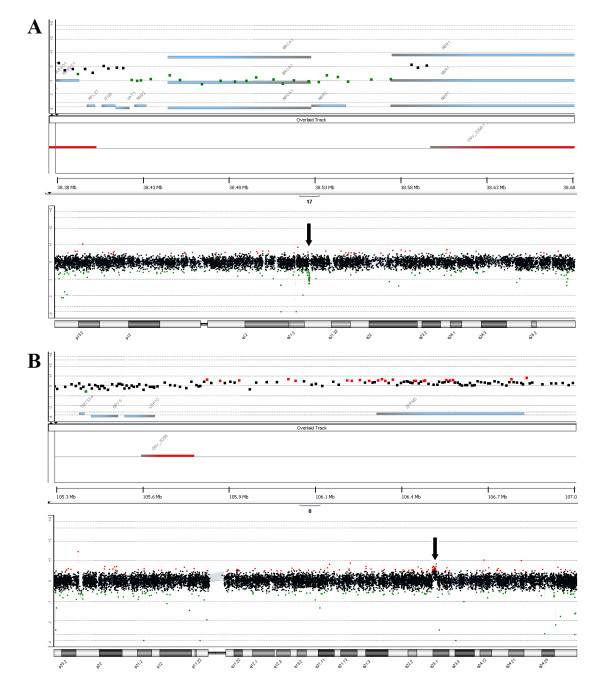
**Representative array CGH profile in the proband**. (A) Schematic representation of Array CGH analysis on chromosome 17. A single-copy deletion from the beginning of *NBR1 *to the *VAT1 locus*, including the *RND2*, *ΨBRCA1*, *BRCA1 *and *NBR2 *complete genes is shown. (B) Schematic representation of Array CGH analysis on chromosome 8. A mosaicism for an amplification of the region corresponding to the *ZFPM2 locus *could be appreciated.

With the aim of characterizing the deleted region, a microarray-based comparative genomic hybridization (CGH) was performed using high-resolution oligonucleotide microarray (Human Genome CGH Microarray 244 K). Oligonucleotide whole genome array CGH analysis showed that the 5' boundary of the affected region is located at the beginning of the *NBR1 *gene whereas the 3' boundary is located around the *VAT1 locus*, including the *RND2*, *ΨBRCA1*, *BRCA1 *and *NBR2 *complete genes with an approximate size of 0.15 Mb (Figure 3A).

**Figure 4:**
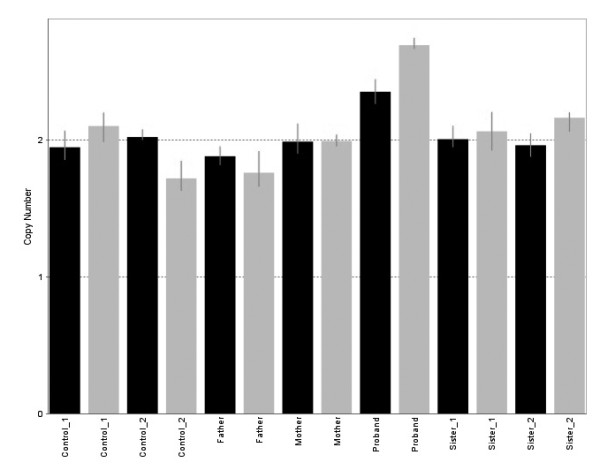
**Copy number of the *ZFPM2 *gene in the proband, her mother, father, two sisters and two control samples**. Each bar represents the copy number prediction of the target sequence in each subject and different colors correspond to each copy number assay (grey: Hs02556672_cn, black: Hs06234652_cn). Thus, each individual is represented by two bars. The broken line indicates the reference line for two copies.

Moreover, another chromosomal abnormality was detected in the long arm (q) of chromosome 8. Specifically, an amplification of the region corresponding to the *ZFPM2 locus *was detected (Figure 3B). These changes did not correspond to CNV according to Genomic Workbench Standard Edition 5.0. These data were confirmed using two CNA of *ZFPM2 *gene in the proband, her mother, father and two sisters and two control samples. The confidence of prediction for both CNA was greater than 95% in each subject. All the samples were predicted to have 2 copies of the *ZFPM2 *gene by any assay excepting the proband sample, which was predicted to have 2.68 and 2.37 copies of the target sequence by assays at the 5'-end of the gene and the 3'-end respectively (Figure 4). Therefore, this subject is likely to have more than 2 gene copies.

## Discussion

In the present study, we describe a *de novo *deletion of *BRCA1 *in the germinal line of an early-onset breast cancer patient. To our knowledge, this represents the third case of a Hereditary Breast and Ovarian Cancer patient with a complete *BRCA1 *gene deletion [19,20]. This rearrangement was detected by MLPA and characterized by Array CGH analysis. The deleted area started from the region surrounding the *VAT1 *(MIM#604631) locus to the beginning of *NBR1 *(MIM#166945) gene, including the *RND2 *(MIM#601555), Ψ*BRCA1*, *BRCA1 *(MIM#113705) and *NBR2 *complete genes. *NBR1 *was originally cloned as a candidate for the ovarian cancer antigen CA125 [21], but no involvement in breast or ovarian cancer has been demonstrated. NBR1 has been described as a highly conserved multidomain scaffold protein involved in targeting ubiquitinated proteins for degradation [22]. No functions have been ascribed to either the Ψ*BRCA1 *or the *NBR2 *genes, which seem to result from a duplication event [23]. Regarding RND2, which is a RHO family small GTPase, this is involved in regulating the migration and morphological changes associated with the development of pyramidal neurons [24]. Finally, *VAT1 *codifies for a synaptic vesicle integral membrane protein [25].

Inadvertently, we also detected an amplification of the region corresponding to the *ZFPM2 locus *(8q23) which did not affect any of the analysed relatives. *ZFPM2 *(MIM#603693) encodes a zinc finger protein member of the FOG family of transcription factors implicated in heart morphogenesis and cardiogenesis. Defects in this gene may be a cause of tetralogy of Fallot (TOF), a congenital heart anomaly, and are also the cause of a form of congenital diaphragmatic hernia (CDH). However, our patient did not show any clinical evidence in this regard. *BRCA *genes are involved in the repair of DNA double-strand breaks (DSBs) by homologous recombination maintaining the genetic stability during cell division. In the absence of functional BRCA1 or BRCA2 DSBs are repaired by an error-prone non-homologous end-joining mechanism that provokes mutations and genomic instability [26]. The case herein reported is deficient in *BRCA1 *and we may speculate that it would be prone to accumulate genetic instabilities that in this case affect the *ZFPM2 *region.

The great peculiarity of the case herein reported is that the *BRCA1 *deletion is not present in any other family member, including both parents. Therefore, it would constitute the first case of a patient with a *de novo *whole-gene *BRCA1 *deletion.

Since the incorporation of LGR analysis into the standard practice of genetic counselling laboratories the number of LGRs reported have almost tripled for *BRCA1 *and sextupled for *BRCA2 *just in the last 4 years, including at least 81 different LGRs in *BRCA1 *[9]. Most of the characterized LGRs in *BRCA1 *have been described throughout the gene as intragenic deletions or duplications resulting from unequal recombination events between *Alu *sequences; the majority are unique, and generally introduce a premature termination codon in the reading frame. This fact is justified by the genetic structure of *BRCA1 *with numerous intragenic *Alu *repeats (41.5%) [25], which are known to mediate the occurrence of rearrangements, and with a *BRCA1 *pseudogene 30 kb upstream [27,28]. Several studies have described germline LGRs involving either *Alu *repeats or the *BRCA1 *pseudogene [29]. However, to our knowledge, only one LGR without involvement of these genetic structures has so far been reported [30].

The frequency of these LGRs in the *BRCA1 *gene varies from 0% in Iranian, Afrikaner and French-Canadian populations, to 27% in the Dutch population [9,31-33]. According to Sluiter and van Rensburg [9] the proportion of LGRs detected in the Hispanic population is over a 10%, probably as consequence of a single founder deletion of exons 9-12 [34]. The size of these *BRCA1 *LGRs varies from 244 bp, the smallest size deleting exon 5 (NG_005905.2:g.111421_111664del) [35], to tens of kilobases removing the complete *BRCA1*gene. As far as we know, there are only two Hereditary Breast and Ovarian Cancer families with LGRs including a whole-gene *BRCA1 *deletion reported to date [19,20]. De la Hoya *et al. *[19] reported a Spanish family (HSP-198) with a complete deletion of *BRCA1 *that segregates with the disease within the family. The alteration was tested by MLPA with the *BRCA1 *P002 probe mix assay and confirmed with the alternative set of probes P087, although no other molecular technique was employed. Moreover, they did not characterize the region affected by the deletion. In 2008, Konecny *et al. *[20] also identified a complete *BRCA1 *gene deletion using a combination of SNP haplotype analysis, MLPA (kits P002 and P0087) and a confirmatory Array CGH analysis. The alteration was also tested to segregate with the disease in the affected family. Excepting these reports, the largest described *BRCA1 *deletion involves 160,880 bp, (NG_005905.2:g.8836_169713del), removing more than 95% of the *BRCA1 *sequence (exons 1-22), Ψ*BRCA1*, *NBR2 *and 18 of the 19 *NBR1 *exons [36].

Regarding the fact that the LGR identified in the proband is a *de novo *mutation, the incidence of *de novo BRCA *mutations among gene carriers is unknown, but seems to be very low. To date, just seven cases of *de novo BRCA *mutations have been reported in the literature, only two in the *BRCA1 *gene and five in *BRCA2 *[37-43] (Table 1). Concerning *de novo *mutations in *BRCA1*, Tesoriero *et al. *[38] report a woman with early-onset breast cancer with two germline protein-truncating mutations: *BRCA1 *c.3769_3770delGA and *BRCA2 *c.5946delT. The *BRCA2 *mutation was inherited from the father and the *BRCA1 *mutation was *de novo *arising also from the father in a testicular germ cell. The second described *BRCA1 de novo *mutation (c.5332+1G > A in intron 21) was reported by Edwards *et al. *[42] in a young woman with early bilateral breast cancer and limited family history. With respect to *de novo *mutations in *BRCA2*, van der Luijt *et al. *[40] identified a *de novo *recurrent germline mutation in *BRCA2 *(c.3034del4) in a patient with early-onset breast cancer and no strong family history of disease, this was the first case with a *de novo *mutation identified in *BRCA2*. In 2002 Robson *et al. *[39] reported a previously undescribed *de novo *mutation in exon 14 of *BRCA2 *(c.7260insA) that results in a premature termination at codon 2359 in a patient diagnosed at 35 with bilateral infiltrating ductal carcinoma and without family history of breast or ovarian cancer (five sisters aged 32 to 47, and mother alive and without cancer at 71). Hansen *et al. *[43] described in 2008 the third case of a *de novo BRCA2 *mutation. This was a novel variant at the splice site of exon 21 (c.8754+1 G > A) in a patient with a ductal carcinoma at the age of 40, with a family history of breast cancer (mother affected at the age of 59), while the novel mutation arose in the male germ line. The two remaining reported mutations were described by Marshall *et al. *[41] and Diez *et al. *[37] in 2009 and 2010 respectively. Marshall *et al. *[41] presented a *de novo BRCA2 *mutation (c.5301insA) in a 35-year-old woman with breast cancer and no strong family history of disease, meanwhile Diez *et al. *[37] identified a novel *de novo BRCA2 *mutation (c.51dupA) in a patient with early-onset bilateral breast cancer and no family history of disease, also located in the paternal allele.

**Table 1:** BRCA1 and BRCA2 de novo mutations.

Gene affected by de novo mutation	Designation*	Clinical Characteristics	Cancer family history	Reference
BRCA1	c.3769_3770delGA	Age < 40 years High-grade BC with axillary nodal metastases.	Father with prostate carcinoma at 50s. Inherited BRCA2 mutation c.5946delT	[38]

BRCA1	c.5332+1G > A	Bilateral IDC BC at 38 (ER+, grade II) and 43 years-old (ER and PR+, grade III).	Maternal aunt with BC prior to her death at 54 years-old.	[42]

BRCA2	c.3034del4	Multifocal BC with axillary node metastases at 39 years of age.	A cousin on the paternal side with BC diagnosed at the age of 54.	[40]

BRCA2	c.7260insA	At age 35 with bilateral IDC.	Father with colon cancer at the age of 57 and died of metastatic disease at 62	[39]

BRCA2	c.8754+1 G > A	IDC BC at the age of 40 (ER and PR +, grade II).	Mother with BC at 59 years-old.	[43]

BRCA2	c.5301insA	At the age of 35 grade III IDC BC (ER+, PR- and HER2-).	Paternal grandmother BC at age 42 and paternal first cousin with prostate cancer at age 40. Maternal family history with diagnosis of OC in great-grandmother and great-great-grandmother, and a great-aunt with BC in her 70s.	[41]

BRCA2	c.51dupA	Diagnosed at the ages of 27 (ER and PR -) and 37 (ER and PR +, HER2 -) with bilateral IDC BC.	No other breast or ovarian cancers were present.	[37]

* Designation of genetic alterations is maintained as previously cited in the original references.

## Conclusions

In conclusion, a relevant number of reports exist of *BRCA *germline mutations in patients with early-onset breast cancer without a strong family history of disease. These studies, including the case herein described, underline the importance of mutation screening, including LGR analysis, especially in cases where tumors are high grade and bilateral. The absence of family history might be related to non-informative families or to the fact that all germ line mutations started as a *de novo *mutation in some ancestor, despite the low incidence of detected *de novo BRCA *mutations.

## List of abbreviations

*CGH*: Comparative Genome Hybridization; *CNA*: Copy Number Assays; *CNV*: Copy Number Variation; *DSBs*: double-strand breaks; *LGR*: Large genomic rearrangement; *MLPA*: Multiplex-ligation-dependent probe amplification.

## Competing interests

The authors declare that they have no competing interests.

## Authors' contributions

JAL, ZG and IR conceived of the study, participated in its design and coordination and helped to draft the manuscript. ZG, AFS and LR carried out the experiments. IR, AG, FL and PL were in charge of clinical data and management of the patient. All authors read and approved the final manuscript.

## Pre-publication history

The pre-publication history for this paper can be accessed here:

http://www.biomedcentral.com/1471-2350/12/134/prepub
